# Characterization and Quantification of Intact 26S Proteasome Proteins by Real-Time Measurement of Intrinsic Fluorescence Prior to Top-down Mass Spectrometry

**DOI:** 10.1371/journal.pone.0058157

**Published:** 2013-03-11

**Authors:** Jason D. Russell, Mark Scalf, Adam J. Book, Daniel T. Ladror, Richard D. Vierstra, Lloyd M. Smith, Joshua J. Coon

**Affiliations:** 1 Department of Chemistry, University of Wisconsin – Madison, Madison, Wisconsin, United States of America; 2 Department of Biomolecular Chemistry, University of Wisconsin – Madison, Madison, Wisconsin, United States of America; 3 Department of Genetics, University of Wisconsin – Madison, Madison, Wisconsin, United States of America; 4 Genome Center of Wisconsin, University of Wisconsin – Madison, Madison, Wisconsin, United States of America; California Institute of Technology, United States of America

## Abstract

Quantification of gas-phase intact protein ions by mass spectrometry (MS) is impeded by highly-variable ionization, ion transmission, and ion detection efficiencies. Therefore, quantification of proteins using MS-associated techniques is almost exclusively done after proteolysis where peptides serve as proxies for estimating protein abundance. Advances in instrumentation, protein separations, and informatics have made large-scale sequencing of intact proteins using top-down proteomics accessible to the proteomics community; yet quantification of proteins using a top-down workflow has largely been unaddressed. Here we describe a label-free approach to determine the abundance of intact proteins separated by nanoflow liquid chromatography prior to MS analysis by using solution-phase measurements of ultraviolet light-induced intrinsic fluorescence (UV-IF). UV-IF is measured directly at the electrospray interface just prior to the capillary exit where proteins containing at least one tryptophan residue are readily detected. UV-IF quantification was demonstrated using commercially available protein standards and provided more accurate and precise protein quantification than MS ion current. We evaluated the parallel use of UV-IF and top-down tandem MS for quantification and identification of protein subunits and associated proteins from an affinity-purified 26****S proteasome sample from *Arabidopsis thaliana*. We identified 26 unique proteins and quantified 13 tryptophan-containing species. Our analyses discovered previously unidentified N-terminal processing of the β6 (PBF1) and β7 (PBG1) subunit - such processing of PBG1 may generate a heretofore unknown additional protease active site upon cleavage. In addition, our approach permitted the unambiguous identification and quantification both isoforms of the proteasome-associated protein DSS1.

## Introduction

Protein analysis is complicated by sequence deviation from that predicted by the genome (e.g., single nucleotide polymorphisms, alternative splicing) and the 200–300 known dynamic post-translational modifications (PTMs) that occur [1], [2], [3]. Intact protein analysis by top-down mass spectrometry (MS) enables the examination of combinatorial PTMs and the ability to identify splice variants while these biologically important features often remain veiled or ambiguous after proteolysis [4], [5], [6]. Yet, the bottom-up approach - using peptides to infer protein identity - continues to be the primary methodology for high-throughput protein analysis, partly due to the greater emphasis on technology and protocols tailored for peptide analysis. With advances in protein separations, bioinformatics, and MS instrumentation, top-down protein analysis has become more tractable permitting online analysis of complex protein mixtures in much the same manner as peptide analysis [7], [8], [9], [10], [11], [12], [13], [14], [15], [16], [17], [18], [19], [20], [21], [22]. Recent work demonstrated that thousands of protein forms can be identified with state-of-the-art top-down technology allowing comprehensive proteome analysis at the intact protein level [23].

Protein identification and characterization have been the hallmarks of sequencing experiments by MS. However, protein quantification is becoming increasingly important because it is often the changes in protein expression and modification state that drive biological events, not exclusively the presence or absence of a particular protein or proteoform (proteoforms describe the protein products derived from a single gene where each uniquely modified protein is termed a ‘proteoform’) [24]. Despite the advances in the field of top-down MS, methodology for intact protein quantification lags behind the large complement of established quantification technologies for peptide analysis [25], [26]. Many of the standard techniques used for relative and absolute peptide quantification have been adapted for the analysis of intact proteins. These techniques include metabolic and chemical labeling strategies for relative quantification [27], [28], [29], [30], [31] as well methods for absolute quantification of intact proteins by chemical labeling, selected reaction monitoring (SRM), and label-free measurements utilizing accurate mass intensity-based measurements [32], [33], [34], [35], [36], [37]. However, many of these strategies have yet to be advanced beyond proof-of-principle experiments. Regardless of technique and whether the goal is to achieve relative or absolute quantification, nearly all require direct measurement of protein abundance by the mass spectrometer. Yet, measurement of gas-phase intact protein ions by MS is complicated by highly variable, and largely unpredictable, ionization, transmission, and detection efficiencies [38], [39], [40]. Accurate quantification of even simple mixtures of dissimilar proteins is a challenge given the nature of large biomolecule ionization and detection. In addition, top-down tandem MS (MS/MS) operated in a data-dependent fashion during liquid chromatography (LC) can suffer from large instrument duty cycle penalties due to the amount of signal averaging required to produce spectra of sufficient signal-to-noise for precursor selection and product ion assignments. Limited MS sampling across a peak during chromatographic elution negatively impacts the reliability of quantification by intensity-based strategies. Because of the factors hindering intact protein quantification by MS, we postulated that the label-free measurement of proteins in the solution-phase prior to MS analysis could provide an improved means for relative protein quantification for top-down MS/MS.

Spectroscopic and fluorometric assays are common strategies to estimate protein abundance, but are not routinely performed on-line during mass spectrometric analysis at nanoflow rates. Native, or intrinsic fluorescence, is an appealing option for biomolecule detection because it is an established, sensitive technique requiring no sample manipulation or labeling chemistry – an important consideration given the diverse chemical properties of complex protein mixtures. Instead, detection relies upon naturally occurring fluorescence emission produced by many biomolecules when excited by ultraviolet light including both proteins and nucleic acid polymers.

Recently, we described the design of an ultraviolet light-induced intrinsic fluorescence (UV-IF) excitation and detection device and methodology permitting the measurement of intrinsic fluorescence in parallel with tandem mass spectrometry during nanoflow chromatographic analysis [41]. We incorporated a fluorescence cell into the fused-silica separation column with integrated electrospray emitter for seamless integration into our nano-flow separation scheme. Our analysis showed relative quantification of tryptophan-containing peptides using UV-IF was often less variable than using peak areas derived from MS signal. Here, we employ the UV-IF-MS/MS strategy for intact protein quantification and provide proof-of-principle of its quantitative value in top-down workflows by providing a full analysis of tryptophan-containing intact protein standards. To demonstrate the utility of parallel top-down MS/MS and UV-IF measurements for protein characterization *and* relative quantification of subunits from a biologically important molecular machine, we applied this technique to analyze intact proteins from an affinity-purified 26****S proteasome complex from Arabidopsis.

## Materials and Methods

### Chemicals and Reagents

All standard proteins and other reagents were acquired from Sigma unless otherwise specified.

### Standard Proteins

Standard proteins (Table S1) were dissolved in 50 mM pH 7.2 phosphate buffer to make 100 pmol/µL stock solutions and stored at 4°C. Working solutions were diluted from the stock solution and used for immediate analysis.

### Arabidopsis Thaliana 26****S Proteasome Affinity Purification

The procedure used to genetically modify and purify 26****S proteasome complexes from *A. thaliana* (ecotype Columbia-0) is described in detail elsewhere [42]. Briefly, the C-terminus of PAG1, a protein subunit in the α-ring of the 20****S core particle, was genetically-modified with the sequence KGGRADPAFLYKVVDYKDDDK containing a FLAG-tag (DYKDDDK). Total protein was extracted from 10-day-old Arabidopsis seedlings containing the PAG1-FLAG tag by extraction of frozen seedlings followed by clarification by centrifugation to remove cellular debris. The soluble homogenate was passed over an affinity column containing beads conjugated with anti-FLAG antibodies. Bound protein was washed with 800 mM NaCl and eluted with an excess of FLAG peptide. Proteins eluted from the affinity column (predominantly from the 20****S core particle) were buffer exchanged against 50 mM ammonium bicarbonate (pH 7.8) and concentrated using 10 kDa nominal molecular weight cut-off centrifugation filters (Millipore Corp.). An estimated 0.5 µg of protein was loaded onto an LC column for analysis.

### Chromatographic Conditions

Micro-capillary columns containing an integrated detection cell and ESI emitter were constructed as previously described [41]. Fused silica tubing (360 µm o.d.×200 µm i.d.) was used to prepare analytical columns slurry-packed to 15 cm in length with Magic C18AQ, 5****µm, 300 Å particles (Michrom Bioresources Inc., Auburn, CA). Precolumns were constructed from 360 µm o.d. ×200 µm i.d. fused silica with a cast chemical frit and slurry-packed with 8–10 cm of the same reversed-phase material [43]. Chromatographic elution was achieved using a nanoACQUITY Ultra Performance LC® system (Waters Corporation, Milford, MA) at 500–750 nL min^-1^ analytical flow rates. Mobile phase A consisted of 0.2% formic acid while mobile phase B contained 99.8% ACN/0.2% formic acid, respectively. Sample concentration/desalting onto the precolumn was carried out at 2.0 µL min^-1^ for 5 min with 5% mobile phase B. Gradients were optimized based on sample type and complexity, but generally consisted of a linear increase in % mobile phase B starting with 10–15% B and increasing at rates of 0.25–1% B per minute.

### Top-down MS and MS/MS

An LTQ Orbitrap Velos enabled for electron-transfer dissociation (ETD - Thermo Scientific, Bremen, Germany) was used for all MS and MS/MS analyses [18], [44]. Orbitrap (Fourier transform, FT) automatic gain control (AGC) target values for MS and MS^n^ scans were 1E6 and an MS AGC target of 4E4 was used for quadrupole linear ion trap (QLT) MS. For chromatographic analyses of standard proteins, MS/MS was not performed, in order to maximize the number of MS scans collected during elution. For experiments using FT-MS/MS, activation time was set at 10 ms for higher-energy collisional dissociation (HCD) with normalized collision energies of 26–30 V. ETD activation was varied from 5–25 ms with an anion AGC target value of 3.0E5. Orbitrap resolution for MS and MS/MS experiments were set at 100****k and 60****k, respectively. The most intense precursors, excluding +1, +2, and +3, were interrogated in a data-dependent manner with back-to-back ETD and HCD scans on the same precursor. Dynamic exclusion was enabled. Maximum fill times were set at 1000 ms for MS and 2500 to 3500 ms for MS/MS scans. Microscans were set at 1–3 for MS and 3–7 for MS/MS.

### Automated Database Searching

Automated database searching was performed using ProSightPC 2.0 (Thermo Fisher). [13], [45] ETD and HCD spectra were converted to monoisotopic masses using the Xtract algorithm and searched individually against a custom Uniprot database for *Arabidopsis thaliana* (mouse-ear cress) created using the Database Wizard in ProSightPC 2.0. The database contained 29,472 basic protein sequences with 565,220 protein forms. The endogenous peptides option was selected. Searches were performed by first using the Absolute Mass search mode with a 3.0 Da precursor and 15 ppm fragment ion tolerances using monoisotopic masses. The minimum number of matching fragments to be considered for identification was set to 10. Spectra that failed to produce a protein-spectrum match or matches with E-value scores less than 10^−4^ were searched in Absolute Mass mode with a precursor tolerance of 10,000 Da, 15 ppm fragment ion tolerance, with the Δm option enabled [46]. Spectra failing to produce an identification or producing matches with E-value scores less than 10^−4^ were searched in Biomarker mode with 10 ppm precursor and fragment ion tolerances with a minimum of 7 fragment ions for a protein-spectrum match. Include Modified Forms was enabled along with the Δm option in Biomarker mode. Finally, protein-spectrum matches with E-values less than 10^−4^ or with mass errors greater than 2 Da were searched in Biomarker mode in an attempt to find the exact subsequence of the protein. Protein sequence coverage was first computed for both ETD and HCD and later combined to yield total sequence coverage.

### Fluorescence Excitation and Detection Device

A full description of the device, its performance, and analytical figures of merit can be found elsewhere [41]. Briefly, a capillary LC column, fabricated with a detection cell and integrated electrospray tip, was mounted to a breadboard with the capillary detection cell positioned above an AlInGaN UV-LED (max λ_em_ = 285 nm) with an integrated ball lens (Sensor Electronic Technology, Columbia, SC) and a bandpass interference filter (Semrock, Rochester, NY) with a center wavelength of 280 nm and a bandwidth of 20 nm. A second fused silica ball lens with a diameter of 4 mm (ISP Optics, Irvington, NY) was placed between the LED and the capillary such that UV-IF was collected 90° relative to the excitation light. Collected UV-IF was filtered using a longpass colored-glass filter with a cut-on wavelength of 324 nm (Newport) followed by a bandpass interference filter with a center wavelength of 357 nm and a bandwidth of 44 nm (Semrock). The breadboard supporting the entire system was mounted on a 3-D translation stage to allow positioning at the MS inlet.

## Results and Discussion

### Analysis of Standard Proteins

**Figure 1:**
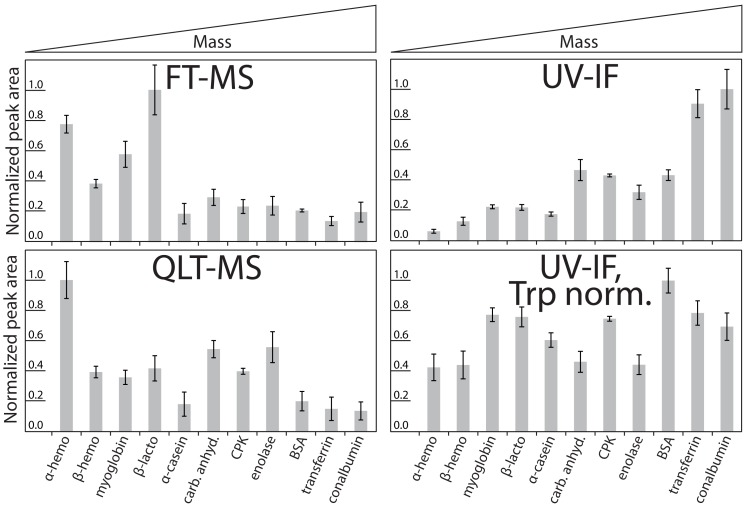
Chromatographic analyses of Trp-containing standard proteins with detection by UV-IF, QLT-MS, or FT-MS. An estimated 1 pmol of protein was chromatographed individually and signal response (peak area) for each protein at each detector was normalized. Proteins are arranged on the graph by increasing average molecular mass (α-hemoglobin = 15.2 kDa, 1 Trp; β-hemoglobin = 16.0 kDa, 2 Trp; myoglobin = 17.1 kDa, 2 Trp; β-lactoglobulin = 18.4 kDa, 2 Trp; α-casein = 24.5 kDa, 2 Trp; carbonic anhydrase = 29.1 kDa, 7 Trp; creatine phosphokinase = 43.1 kDa, 4 Trp; enolase = 46.8 kDa, 5 Trp; bovine serum albumin = 69.3 kDa, 3 Trp; transferrin = 77.1 kDa, 8 Trp; conalbumin = 77.8 kDa, 10 Trp). The Trp-normalized UV-IF response (bottom right) was determined by dividing the UV-IF response (top right) by the number of Trp residues in each protein’s primary sequence. Error bars represent 2 standard deviations.

Intrinsic fluorescence from proteins and peptides excited by 280 nm UV light is dominated by emission from tryptophan, a relatively rare amino acid accounting for less than 2% of the amino acids in the proteomes of many model organisms [47]. In the case of *Arabidopsis thaliana*, tryptophan represents 1.3% of amino the acids in its proteome. However, nearly 88% of the proteins in the *A. thaliana* proteome contain at least one tryptophan residue. The fluorescence excitation and detection device was designed to maximize the detection of fluorescence emission from tryptophan while minimizing fluorescence contributions from tyrosine and phenylalanine [41]. We used this device to assess the concentration-dependent response of standard proteins (Table S1) ranging in molecular mass from 15–78 kDa containing 1–10 tryptophan residues. An estimated 1 pmol of each protein was individually chromatographed in triplicate using an identical 40-minute gradient. Peak areas were integrated and normalized to UV-IF, QLT-MS, and FT-MS signal responses. For MS analyses, peak areas from extracted ion chromatograms (+/−2.5 Th) corresponding up to the 25 most abundant charge states spanning a range of *m/z* 400–2000 were summed. Signal response, arranged by increasing protein molecular mass, is shown in Figure 1. Protein UV-IF response increased with increasing protein mass (Figure 1, upper right); while, QLT-MS (quadrupole linear ion trap, upper left) and FT-MS (orbitrap, lower left) response generally decreased with increasing protein mass. Relative UV-IF response also increased with increasing numbers of Trp residues as the higher mass proteins used in this study, as in nature, generally have a greater number of tryptophan residues. These observations agree with previous reports of UV-IF of intact proteins [48].Decreasing MS signals for both mass analyzers were observed for proteins of increasing mass and may be attributed to several factors including low ionization and transmission efficiencies, as well as diminished detector signal due to decreased ion-to-electron conversion efficiency (QLT) or ion cloud dephasing associated with gas-phase collisional cross sections (FT) [38], [39], [40], [49], [50], [51], [52], [53].

**Figure 2:**
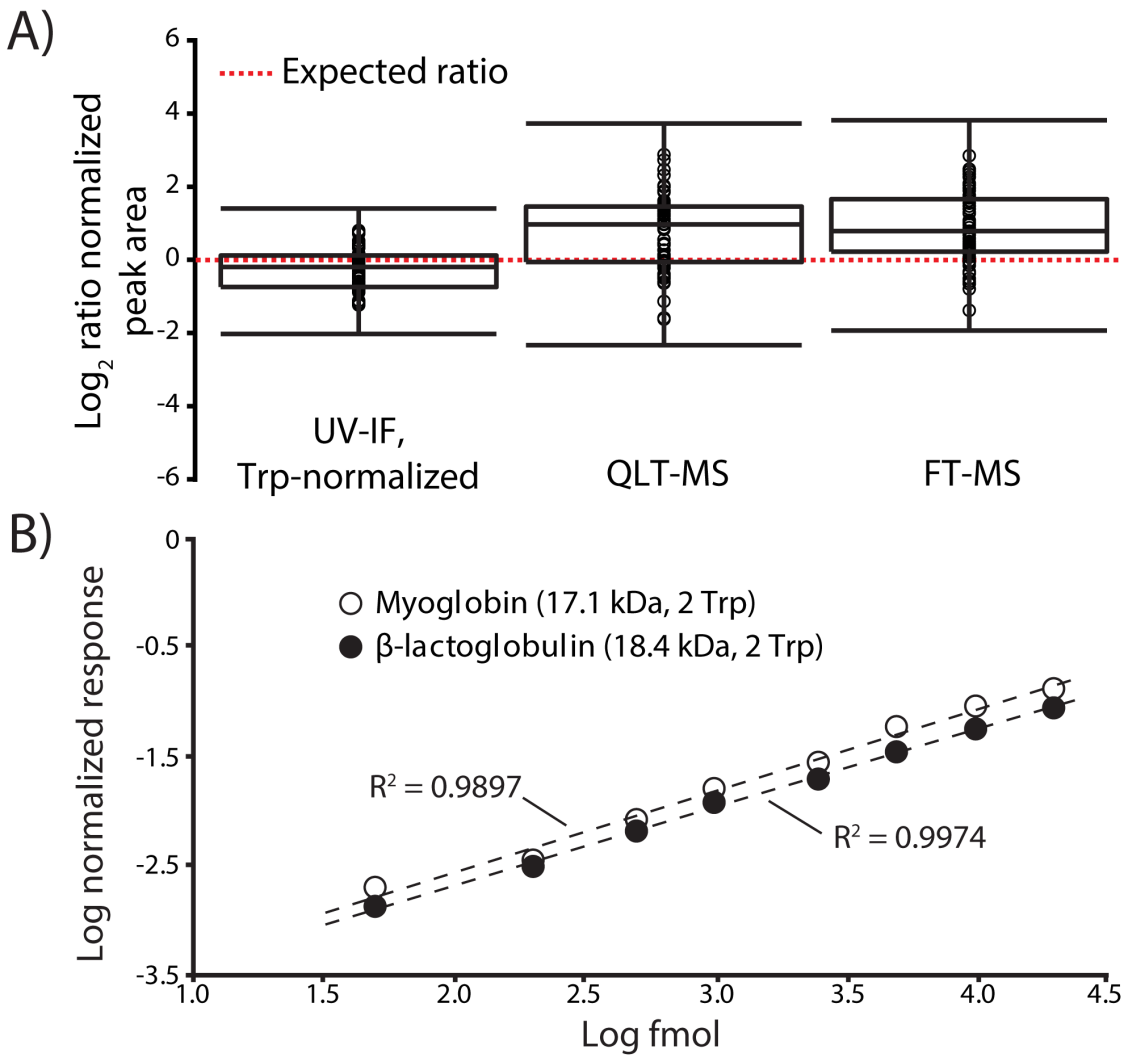
Quantification accuracy, precision, and dynamic range. Aliquots of 11 proteins resulting in 1 pmol on-column amounts were individually chromatographed in triplicate. The signal response (peak area) for each protein was normalized relative to each detector and the ratios of protein responses were plotted on a log_2_ scale. (**A**) Trp-normalized UV-IF provides better quantification accuracy and precision for the analyses of Trp-containing protein standards. (**B**) The UV-IF concentration-dependent response is linear over 3 orders of magnitude (20 fmol to 20 pmol on column) for myoglobin and β-lactoglobulin.

These data suggest that fluorescence signal scales with the number of tryptophan (Trp) residues in a protein sequence; therefore, normalization of UV-IF signal to the number of Trp residues should produce similar UV-IF responses for most Trp-containing proteins. Trp normalization produced UV-IF signals with no clear bias towards molecular mass (Figure 1, lower right). Ideally, there should be a one-to-one protein response as we estimated that equimolar amounts of protein were loaded on the separation column. However differences in protein purity, solubility, and each protein’s amenability to be chromatographed under reversed-phase conditions contributed deviations from theoretical estimations. In addition, natural variation in UV-IF response is expected due to the sensitivity of protein UV-IF to local environment (e.g., temperature, solution polarity, pH) and degree of protein denaturation. UV-IF sensitivity to local environment is especially pronounced for residues buried within a protein. However, many proteins partially to fully denature during reversed-phase gradient elution leading to linear and often predictable fluorescence responses [54], [55]. Previous reports have shown that the fluorescence quantum yield from tryptophan-containing proteins can be predicted with a high degree of certainty when the effects of environmental variables can be reliably modeled [56]. We believe the effects of local environment on UV-IF during chromatographic analyses to be minor; however, more investigation into these effects is warranted. Despite the above mentioned caveats, the use of Trp-normalized UV-IF was both more accurate and more precise for intact protein quantification compared to either mass analyzer (Figure 2A). As the data in Figure 2A indicate, there are no outliers in the datasets collected on standard proteins despite the fact that there is a large range in normalized peak area values for the MS results. However, if the most and least abundant proteins measured by each detector were omitted from the analysis, relative quantification by UV-IF still provides improved quantification over MS. The normalized peak area values span a range 1.8-fold for Trp-normalized UV-IF and greater than 3.7- and 4.1-fold for QLT-MS and FT-MS, respectively. In addition to quantification accuracy and precision, other important figures of merit for quantification by UV-IF include a linear dynamic range of over 3 orders of magnitude (Figure 2B) with on-column limits of detection approaching 5 fmol and limits of quantification near 20 fmol. These analytical figures of merit rival any current intact protein quantification technology relying upon chromatographic separations and mass spectrometric measurements.

**Figure 3:**
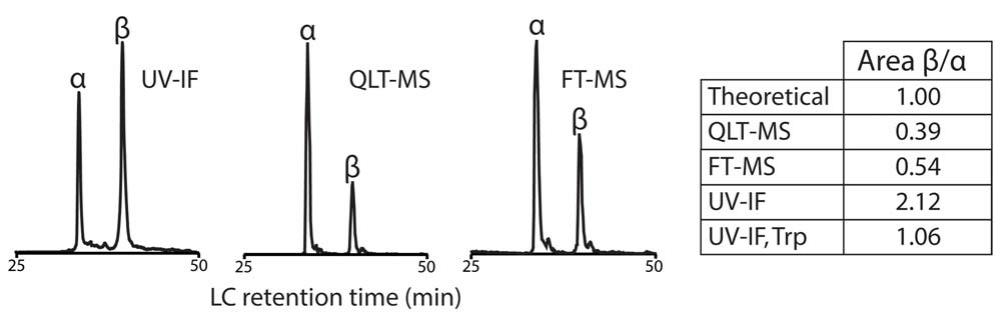
Chromatographic analysis of hemoglobin validates signal normalization by the number of Trp residues. Hemoglobin is a heterotetramer which dissociates during eluting producing equimolar amounts of α-(15.2 kDa) and β-(16.0 kDa) subunits containing 1 and 2 Trp residues, respectively. The Trp-normalized UV-IF response was determined by dividing the UV-IF response by the number of Trp residues in each subunit. UV-IF produced a near 1∶1 ratio; while both mass analyzers underestimated the relative amount of the β-subunit by ∼ 50%.

To further validate normalization of UV-IF signal to number of tryptophan residues we analyzed bovine hemoglobin. Bovine hemoglobin is a protein heterotetramer comprising two α-(15.2 kDa) and two β-(16.0 kDa) subunits each noncovalently attached to a heme group. The α- and β-subunits contain one or two tryptophan residues, respectively. During reversed-phase gradient elution, noncovalent interactions in hemoglobin can be disrupted and the α- and β-subunits may be separated producing on-column equimolar amounts of hemoglobin subunits containing one and two tryptophan residues, respectively (Figure 3). The peak area ratio of β:α is expected to be approximately 1; however, QLT-MS and FT-MS produced ratios of 0.39 and 0.54, respectively. With UV-IF, the normalized ratio of β:α was 1.06. These data, considered with the entire analysis of standard proteins, demonstrates that Trp-normalized UV-IF protein quantification produces results closer to prediction than either mass analyzer.

The α- and β-subunits were chromatographically resolved in this example, a prerequisite for quantification of protein mixtures using UV-IF. Because of the chromatographic demands on separation, this quantification strategy will be most applicable protein mixtures of low-to-moderate complexity (e.g., purified protein complexes) where chromatographic conditions can be optimized to achieve the greatest chromatographic resolution. To demonstrate the utility of UV-IF quantification in beyond standard proteins, we applied this technology along with high mass accuracy tandem mass spectrometry to identify and quantify proteins of a biologically relevant molecular machine.

### Analysis of *A. thaliana* 26****S Proteasome

The 26****S proteasome is a 2.5 MDa protein complex responsible for degradation of ubiquitinated proteins in cells. The 19S regulatory particle (RP) and 20 S core particle (CP) comprise the 26S proteasome and both the 19S RP and 20 S CP are formed by the assemblage of proteins (subunits) with documented stoichiometries [57]. However, there are a number of subunit isoforms and neither their stoichiometries, nor the biological implications of isoform substitutions, are known. In previous work, we performed shotgun analyses on affinity-purified 26S proteasome complexes from *A. thaliana* identifying ∼50 proteins from each purified sample with the majority of the identifications coming from subunits of the 20 S CP (∼25–30 kDa), and 19S RP (∼45–50 kDa) [42]. We reasoned this sample would be ideally-suited to test our UV-IF MS/MS approach because it was both relatively low in complexity, well characterized, and contained subunits <30 kDa easing the demands on LC separation and MS/MS performance. Enrichment for 20 S CP proteins was achieved through the use of salt washes which released the large and difficult to chromatographically separate 19S RP proteins and also removed loosely associated proteins and proteins non-specifically bound to the affinity column. The sample was separated over a 90-minute reversed-phase gradient using back-to-back ETD and HCD MS/MS analyses of the same precursor. Because quantification would be achieved by UV-IF, we limited the number of MS1 scans and instead invested more duty cycle towards collecting high-quality MS/MS spectra performing up to 7 microscans (transient averaging) for each back-to-back ETD/HCD scan. From a single analysis, 193 protein-spectrum matches were made with E-values less than 5.29E^-05^ corresponding to 26 unique protein accession numbers (Table 1). At least one isoform from every 20 S CP α- and β-type subunit was identified using MS/MS with the exception of PAF1 or PAF2. Nearly every unique protein was identified from multiple charge states giving us increased confidence in sequence assignments. Of the 26 high-scoring protein identifications, 22 contained at least one tryptophan residue.

**Table 1:** Proteins identified from a single UV-IF top-down MS/MS analysis of an affinity-purified 26S proteasome sample.

**Protein description**	**Accession**	**Gene name**	**Product ions**	**Theoretical** **mass (Da)**	**Observed mass (Da)**	**ΔMass (ppm)**	**MS/MS type**	**PTMs (position)**	**E-value**	**Trp**
DSS1-1	Q9XIR8	DSS1(I)	110	8764.943	8765.004	7.0	ETD	N-acetyl-L-alanine (1)	9.13E−136	3
PSA4	O81148	PAC1	77	27368.831	27368.981	5.5	ETD	N-acetyl-L-serine (1)	6.53E−112	2
DSS1-2	Q9FL96	DSS1(V)	79	8603.978	8604.023	5.2	ETD	N-acetyl-L-alanine (1)	4.31E−100	3
PSA6B	O81147	PAA2	55	27243.818	27243.954	5.0	ETD	N-acetyl-L-serine (1)	3.27E−90	1
PSA6A	O81146	PAA1	58	27187.769	27187.899	4.8	ETD	N-acetyl-L-serine (1)	5.23E−88	1
PSA5B	Q42134	PAE2	54	26002.965	26002.981	0.6	ETD	N-acetyl-L-methionine (1)	1.01E−82	1
PSA7A	P30186	PAD1	55	27231.354	27231.499	5.3	ETD	N-acetyl-L-alanine (1)	6.09E−82	1
PSA5A	O81149	PAE1	54	25972.954	25973.043	3.4	ETD	N-acetyl-L-methionine (1)	7.86E−78	1
PSB2B	O24633	PBD2	74	22012.187	22012.155	-1.5	ETD	N-acetyl-L-methionine (1)	2.83E−77	1
PSB1	P42742	PBF1	62	24003.019	24003.067	2.0	ETD	N-terminal processing (removal of 1–5)	6.21E−77	1
PSB5A	O23717	PBE1	55	23367.374	23367.354	−0.9	ETD	N-terminal processing (removal of 1–57)	3.21E−73	3
PSB3A	Q9XI05	PBC1	54	22694.464	22694.574	4.8	ETD	N-acetyl-L-serine (1)	8.90E−70	1
PSB4	Q7DLR9	PBG1	50	25067.758	25067.781	0.9	ETD	N-terminal processing (removal of 1−23)	4.59E−67	5
RL391	P51424	RPL39C	41	6280.545	6280.567	3.5	ETD		2.01E−64	2
PSB7A	O23710	PBB1	43	25323.842	25322.926	−36.2	ETD	N-terminal processing (removal of 1–39)	7.23E−62	0
PSB2A	O23714	PBD1	53	22568.472	22568.576	4.6	ETD	N-acetyl-L-methionine (1)	1.46E−61	1
PSB5B	Q9LIP2	PBE2	41	23372.416	23372.501	3.6	ETD	N-terminal processing (removal of 1–57)	9.47E−57	3
PSB6	Q8LD27	PBA1	45	23854.001	23854.125	5.2	ETD	N-terminal processing (removal of 1–12)	1.58E−54	3
PSA2A	O23708	PAB1	36	25596.246	25596.338	3.6	ETD	N-acetylglycine (1)	1.30E−50	1
PSA2B	Q8L4A7	PAB2	35	25628.236	25628.266	1.2	ETD	N-acetylglycine (1)	1.31E−50	1
PSB3B	O81153	PBC2	33	22655.480	22655.622	6.3	ETD	N-acetyl−L-serine (1)	6.03E−50	1
PSA3	O23715	PAG1	37	29764.970	29765.076	3.6	ETD	N-acetyl-L-serine (1)	3.28E−47	3
PSB7B	Q7DLS1	PBB2	25	25385.906	25385.966	2.4	ETD	N-terminal processing (removal of 1–39)	1.78E−37	0
RLA22	Q9SLF7	RPP2B	17	11516.816	11516.857	3.6	ETD	O-phospho-L-serine (105)	1.02E−22	0
CYT1	Q945Q1	CYS1	26	11159.650	11159.685	3.1	HCD	N-acetyl-L-alanine (1)	6.67E−21	1
PLAS2	P42699	DRT112	19	10445.076	10445.147	6.8	HCD	N-terminal processing (removal of 1–68)	1.66E−10	0

Listed masses are monoisotopic values.

Trp = number of tryptophan residues sequenced. The results reported in this table are from a single analysis.

**Figure 4:**
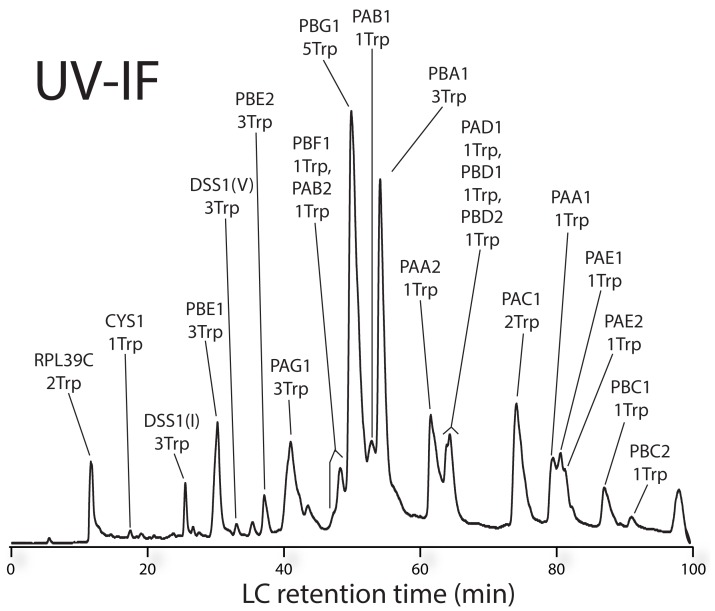
UV-IF chromatogram of affinity-purified 20 **S proteasome sample from** ***A. thaliana*** **.** Trp-containing proteins identified using MS/MS with assignment to UV-IF peaks.

**Figure 5:**
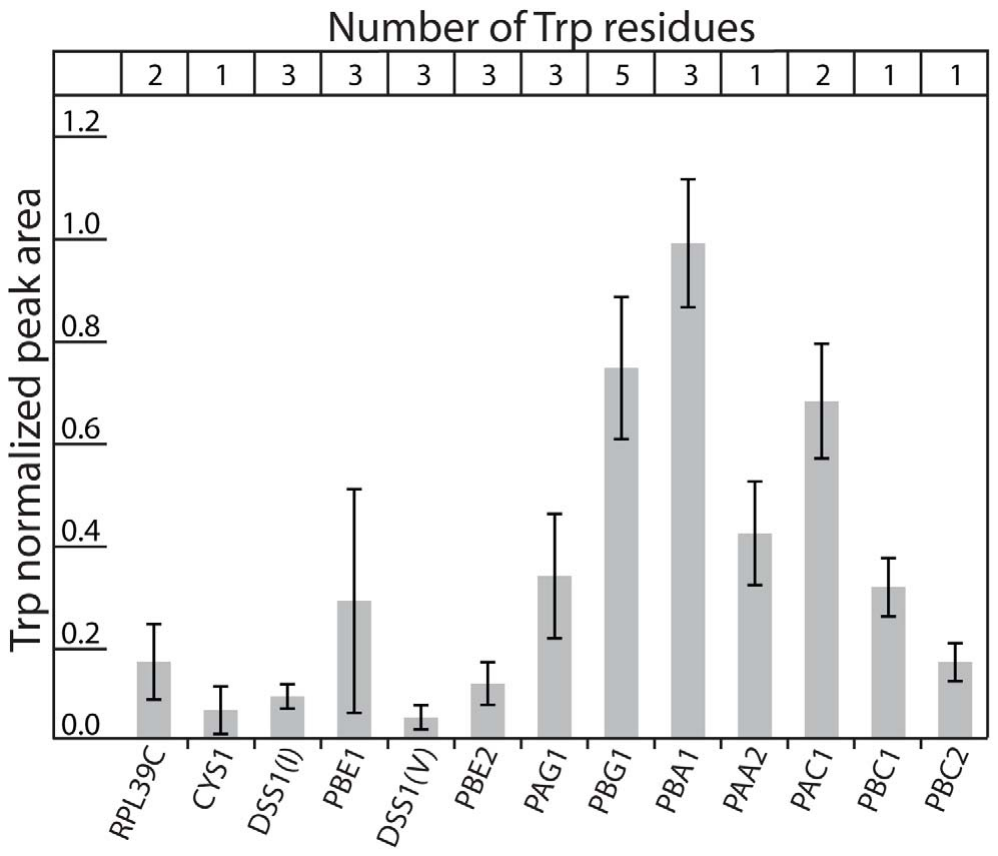
Quantification of Trp-containing proteasome subunits. Proteins exhibiting UV-IF identified from the affinity-purified 20 S proteasome sample (*A. thaliana*) were quantified based upon their peak areas (FWHM) normalized to the number of Trp residues in each protein. In total, 13 Trp-containing proteins were chromatographically resolved to permit quantification from the analysis of 3 separate affinity purifications (N = 3).

Proteins were assigned to UV-IF peaks (Figure 4) using the retention time of the protein identification and precursor mass (10 ppm mass tolerance) as guides. UV-IF peak areas were estimated by measuring the peak full width at half maximum (FWHM) utilizing a Gaussian peak shape approximation followed by tryptophan signal normalization [58]. Proteins for which peak areas could not be accurately estimated due to insufficient resolution or for which spectral evidence suggested a co-eluting species were excluded for quantification. In the analysis of 3 independent proteasome purifications of *Arabidopsis* seedlings (biological triplicates), 13 tryptophan-containing proteins were sufficiently resolved chromatographically to permit relative quantification based on UV-IF (Figure 5, Figure S1). Of these 13 proteins, 9 are known constituents of α- and β-rings of the 20 S CP [42], [59]. The most abundant protein based upon UV-IF was the subunit PBA1 followed by PBG1 and PAC1 which were 0.75 and 0.68 times as abundant as PBA1. These values are in good agreement with gene expression values generated from expressed sequence tags (ESTs) from *A. thaliana* where PBG1 and PAC1 were 0.87 and 0.99 times as abundant as PBA1 [42]. Our analysis revealed extensive N-terminal modification of the 20 S CP subunits. Every identified subunit had either N-terminal acetylation or processing of the N-terminus (propeptide cleavage) to produce mature proteoforms. The β5 subunit isoforms PBE1 (Figure S2) and PBE2 (Figure S3) were identified from our bottom-up dataset, yet direct evidence of the predicted N-terminally cleaved proteoforms could not be established after proteolysis. Using top-down MS, we identified both PBE1 and PBE2 (92% sequence homology) and confirmed cleavage between Gly^57^ and Thr^58^ on both isoforms. We determined that PBE1 was ∼2.5 times more abundant than PBE2 by UV-IF similar to EST results where PBE1 was 3.4 times more abundant than PBE2 [42]. Processing to expose an N-terminal threonine imparts catalytic activity to PBE1 and PBE2 when incorporated as β5-subunits of the 20 S CP. The biological consequence(s) of differential incorporation of these isoforms at the β5 position is not known.

### The Use of Multiple Dissociation Techniques Maximizes Protein Sequence Coverage

The highest-scoring protein identifications were typically generated from ETD, yet HCD also produced quality MS/MS spectra. The strategy of using ETD and HCD in consecutive MS/MS events proved to be particularly advantageous for maximizing sequence coverage on larger proteins (>20 kDa). For example, both ETD and HCD enabled identification of the N-terminally processed form of PBA1 (23.9 kDa), the most abundant subunit by UV-IF, with E-value scores of 7.55 E^-55^ and 4.48E^-28^ with 20.5% and 20.1% sequence coverage, respectively (Figure S4). Total sequence coverage of PBA1 was increased to 36.4% by considering both dissociation techniques. The use of both ETD and HCD facilitated the confident identification of novel N-terminal processing of the 20 S CP subunit PBF1 (Figure S5). Cleavage of a portion of the N-terminus of PBF1 in Arabidopsis was predicted to occur based upon sequence homology with yeast; yet, evidence of this PTM in Arabidopsis has not been reported at the protein level [42], [59]. The yeast ortholog of PBF1(PRS3/C5) is cleaved between the His^19^ and Glu^20^ residues [60]. Through the use of the biomarker search mode in ProSightPC, we localized N-terminal cleavage between the His^5^ and Ala^6^ residues (2.0 ppm mass error). ETD generated abundant fragment ions from the C-terminal portion of the protein, but very little N-terminal coverage. HCD, however, generated a 7-residue sequence tag and produced 12 product ions from the first 18 residues of the processed N-terminus complementing the ETD result and permitting confident assignment of this PTM. Total sequence coverage for the modified form was 46% using both dissociation techniques.

We also present the first protein-level evidence in Arabidopsis of N-terminal processing of the β7 subunit PBG1 to expose an N-terminal threonine upon removal of the first 23 residues (Figure S6). The β7 subunit of the 20 S CP was observed to be similarly processed in bovine liver where it was proposed by Unno *et al.* to have N-terminal nucleophile hydrolase activity with specificity for small neutral amino acids based upon its amino acid sequence and crystal structure [61]. The potential for catalysis was attributed to Thr^1^, Asp^56^, Arg^99^, and Asn^104^ residues in the bovine β7 subunit which are conserved in β7-subunits of humans. In Arabidopsis, these residues are also conserved in PBG1 with the exception of position 56 where Glu is observed instead of Asp (Figure S7). The targets of proteasome inhibitors - a class of drug compounds for cancer therapy – are most often the catalytic subunits of the 20 S CP [62]. Identification of an additional proteolytic subunit of the 20 S CP could potentially present an additional target for proteasome inhibition. We are currently examining the possible proteolytic activity of PBG1 in Arabidopsis.

### Identification and Quantification of DSS1 Proteins

**Figure 6:**
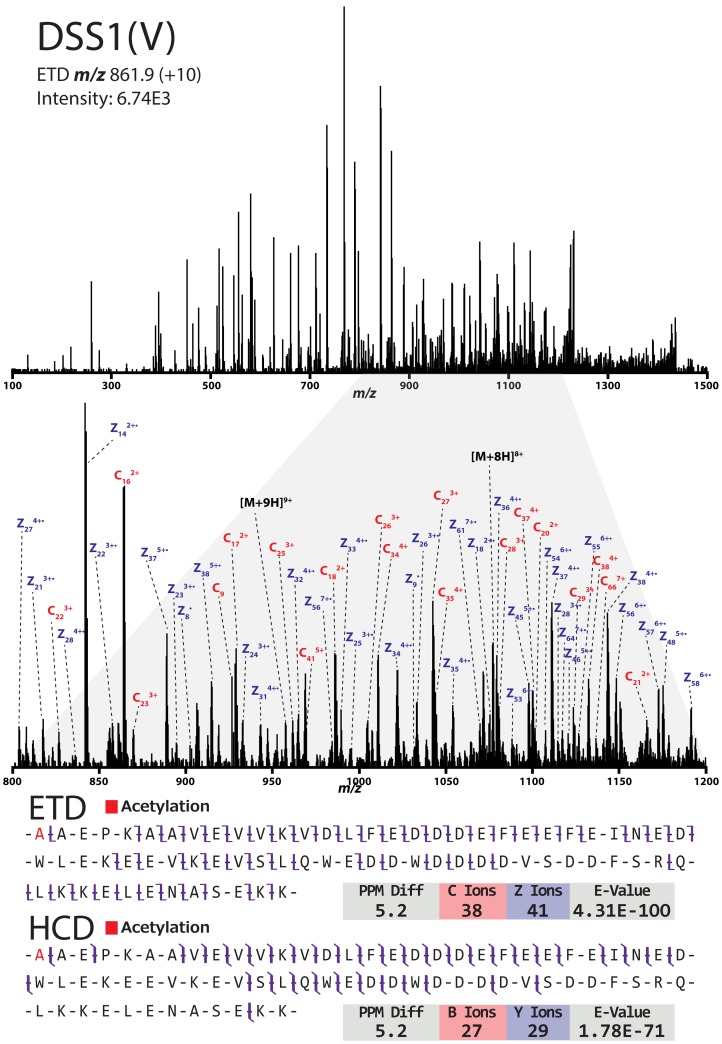
Top-down MS/MS analysis of DSS1(V). ETD spectrum of the proteasome-associated protein DSS1(V) (DSS1-2) and sequence coverage for back-to-back ETD/HCD MS/MS scans. The protein was identified as acetylated on the N-terminal alanine residue.

Analysis of intact proteins facilitated an increase in sequence coverage and revealed important protein forms not identified from our bottom-up data set. Of great interest was the identification of two highly acidic (p.I. ∼ 3.9) proteasome-associated proteins related to mammalian DSS1 and its yeast ortholog Sem1-1 [63], [64]. Prior work with yeast and mammalian cells showed that DSS1/Sem1 bind to the 26S proteasome via direct interactions between DSS1/Sem1 and RPN3 and RPN7 subunits of the 19S RP lid subcomplex [65], [66]. The exact functions of the DSS1/Sem1 family are not yet clear, but their ability to bind both the 26S proteasome and several factors involved in RNA export and genome maintenance suggests that it provides a bridge between the proteasome and various nuclear functions that require protein turnover [67]. In Arabidopsis, DSS1 is encoded by two paralogous loci *Dss1(I)* and *Dss1(V)* that share 87% amino acid sequence identity. DSS1(I) was unambiguously identified from the bottom-up analysis with 34% sequence coverage arising from 3 peptides [42]. Here, we detected intact DSS1(I) with 7.0 ppm precursor mass error obtaining greater than 97% sequence coverage considering both ETD and HCD spectra (Figure S8). Whereas, prior MS after proteolysis failed to unambiguously identify DSS1(V) as a proteasome interacting factor, we identified the intact protein using the top-down approach (5.2 ppm mass error, 81% sequence coverage, Figure 6). Both forms were identified as N-terminally acetylated and each DSS1 protein contains three tryptophan residues permitting quantification by UV-IF. DSS1(I) was two-fold more abundant in this purification relative to DSS1(V) in line with its greater expression levels based on ESTs (1.6-fold) [68]. Challenges to the detection of the DSS1/Sem1 family of proteins by conventional bottom-up LC-MS has been further corroborated by recent investigations into the 19S RP in yeast [69]. The authors were unable to detect endogenous or recombinantly expressed Sem1 protein in purifications of proteasome lid complexes by conventional bottom-up MS. By comparison, all other lid subunit proteins were identified suggesting that a top-down approach, such as that presented here, could offer a complementary strategy the characterization of proteasome subunits in model species in addition to Arabidopsis.

Our finding of the DSS1 proteins in the modified 26S proteasome affinity purification was somewhat surprising. The preparations were enriched for the 20 S CP by washing the 26S complexes with 800 mM NaCl while still bound to the anti-Flag antibody resin. This process selectively releases the 19S RP bound to the 20 S CP which was immobilized through the PAG1-FLAG subunit [42]. It is possible that low amounts of 19S RP proteins remained bound to the affinity column after washing, but were below our limit of detection. Alternatively, the detection of DSS1(I) and DSS1(V) after the salt wash might reflect a strong association of DSS1 proteins with subunits of the 20 S CP in addition to known associations with RPN3A and RPN3B in the 19S RP of Arabidopsis [65]. The specific association of DSS1 proteins with the 26S proteasome, the 20 S CP in particular, is of interest because DSS1 proteins are known interactors with Arabidopsis protein orthologs of BRCA2 (Breast cancer 2 susceptibility protein) as well as components of the nuclear pore [70], [71]. Mutations of the BRCA2 gene are linked to susceptibility to a hereditary form of breast cancer in humans. Detecting both proteins forms and determining their stoichiometry could be especially important given that the two BRCA2 proteins in Arabidopsis, BRCA2(IV) and BRCA2(V), differentially interact with DSS1 proteins. DSS1(V) interacts with both BRCA2(IV) and BRCA2(V) while DSS1(I) interacts exclusively with BRCA2(V) [71]. Our findings suggest a closer examination is needed to determine the extent of DSS1 associations with subunits of the Arabidopsis 26S proteasome with the potential to better understand the interactions between the proteins of BRCA2, DSS1, and the 26S proteasome.

### Conclusions

Determination of intact protein relative abundance using raw MS signal is fraught with difficulty due to dramatic differences in MS signal for protein ions. Highly variable protein response is particularly problematic when attempting to quantify diverse proteins spanning a large mass range (10–80 kDa). The often unpredictable nature of electrospray ionization and ion transmission, combined with technical limitations in modern instrumentation for large biomolecule detection, restricts the utility of MS ion current to provide an accurate estimation of gas-phase intact protein species. The detection of UV-induced intrinsic fluorescence with a nanoLC-compatible fluorescence excitation and detection device provides an orthogonal solution-phase strategy to measure intact protein abundance. We demonstrated that the UV-IF signal response of intact proteins containing tryptophan provides better protein relative quantification than QLT and Orbitrap mass analyzers. The UV-IF signal strongly correlates with the number of tryptophan residues in proteins; thus larger proteins, usually containing more Trp residues, produce more UV-IF signal. This is in direct contrast to gas-phase MS measurements where there is a substantial drop in signal with increasing protein mass. Whereas quantification by MS is not strictly dependent on chromatographic resolution, quantification by UV-IF does require adequate chromatographic resolution to permit reliable estimations of peak areas. This constraint restricts the technique’s applicability to samples of low-to-moderate complexity such as those observed in purified protein complexes. However, as chromatographic separations continue to improve through the use of small diameter particles, ultra-high pressures, and elevated temperatures, we expect this technique to become more applicable to increasingly complex mixtures [72].

However, intact protein analyses by MS are often performed on relatively simple protein mixtures to enable collection of MS/MS spectra of sufficient quality to make an identification. By having a separate detector dedicated to measuring protein abundance, longer MS/MS duty cycles (more transient averaging) can be utilized to produce high-quality spectra for protein sequence analysis. This combined UV-IF MS/MS approach permitted the identification of 26 unique proteins from the affinity-purified 20 S CP of the *A. thaliana* 26S proteasome with relative quantification achieved for 13 protein forms based upon their intrinsic fluorescence. Our strategy of interrogating intact proteins led to the discovery of novel N-terminal processing of PBF1, PBG1, and the unambiguous identification and quantification of both isoforms of the 26S proteasome-associated protein DSS1. These interesting discoveries remained hidden after trypsinization reinforcing the efficacy of intact protein analysis for comprehensive protein characterization.

## Acknowledgments

We are indebted to David Horn (Thermo Fisher Scientific) for help with data analysis using ProSightPC 2.0 software.

## Supporting Information

Figure S1 **Quantification of α- and β-type protein subunits of the 20 S CP.** Subunits that exhibited UV-IF and were sufficiently chromatographically resolved (90-min gradient) to permit peak area approximations were quantified (N = 3). Stoichiometry of each α- and β-type subunit is documented at 1∶1 for the 20****S CP. Affinity purification and quantification by UV-IF produced 20****S CP stoichiometries within a factor of 3 of expected.
(TIF)

Figure S2 **Top-down MS/MS analysis of PBE1.** Fragment ion maps for ETD and HCD fragmentation of the N-terminally processed subunit PBE1 (PSB5A, O23717). This modified form was identified with -0.9 ppm mass error and 38% sequence coverage.
(TIF)

Figure S3 **Top-down MS/MS analysis of PBE2.** Fragment ion maps for ETD and HCD fragmentation of the N-terminally processed subunit PBE2 (PSB5B, Q9LIP2). This modified form was identified with 3.8 ppm mass error and 25% sequence coverage.
(TIF)

Figure S4 **Top-down MS/MS analysis of PBA1.** Fragment ion maps for ETD and HCD fragmentation of PBA1 (PSB6, Q8LD27) suggesting N-terminal processing of the first 12 residues. This modified form was identified with 5.2 ppm mass error and 36% sequence coverage.
(TIF)

Figure S5 **Top-down MS/MS analysis of PBF1.** Fragment ion maps for ETD and HCD fragmentation of PBF1 (PSB1, P42742) suggesting N-terminal processing of the first 5 residues. This modified form was identified with 2.0 ppm mass error and 46% sequence coverage.
(TIF)

Figure S6 **Top-down MS/MS analysis of PBG1.** Fragment ion maps for ETD and HCD fragmentation of PBG1 (PSB4, Q7DLR9) suggesting N-terminal processing of the first 23 residues. This modified form was identified with 0.9 ppm mass error and 34% sequence coverage.
(TIF)

Figure S7 **Sequence alignment of Arabidopsis, human, and bovine β7 subunits of the 20 S CP.** The sequence alignment was performed using the Align program from UniProt on the 20****S CP β7 subunits of Arabidopsis (PBG1, Q7DLR9), human (PSMB4, P28070), and bovine (PSMB4, QT3108) species. All three species release propeptides exposing an N-terminal threonine residue and alignment reveals conserved residues at the 56^th^, 99^th^, and 101^st^ positions (relative to Thr^1^) except for the substitution of Glu for Asp at position 56 for Arabidopsis. These 4 positions are believed to be important for potential proteolytic activity of the β7 subunit [61].
(TIF)

Figure S8 **Top-down MS/MS analysis of DSS1(V).** ETD spectrum of the proteasome-associated protein DSS1(I) (DSS1-1) and sequence coverage for back-to-back ETD/HCD MS/MS scans. The protein was identified as acetylated on the N-terminal alanine residue. Sequence coverage and mass accuracy unambiguously identify DSS1(I) from the closely related DSS1(V) protein.
(TIF)

Table S1 **Standard proteins used for quantification studies.**
(DOCX)
