# Supplementary figures and images for: Characterization and Quantification of Intact 26S Proteasome Proteins by Real-Time Measurement of Intrinsic Fluorescence Prior to Top-down Mass Spectrometry
Source: PLoS One. 2013 Mar 11;8(3):e58157. doi: 10.1371/journal.pone.0058157 (PMC3594244; doi:10.1371/journal.pone.0058157)

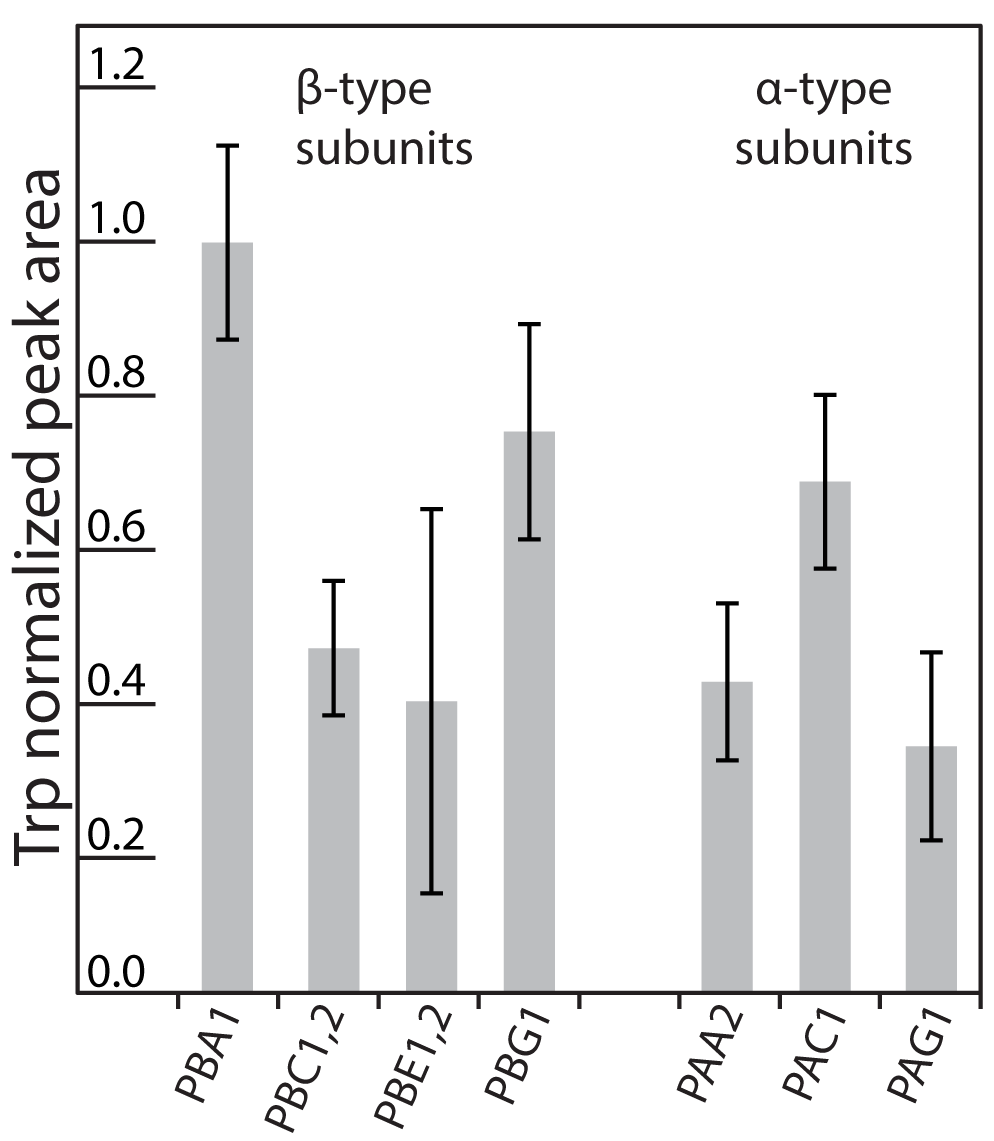

Supplement: Figure S1 — Quantification of α- and β-type protein subunits of the 20 S CP. Subunits that exhibited UV-IF and were sufficiently chromatographically resolved (90-min gradient) to permit peak area approximations were quantified (N = 3). Stoichiometry of each α- and β-type subunit is documented at 1∶1 for the 20S CP. Affinity purification and quantification by UV-IF produced 20S CP stoichiometries within a factor of 3 of expected. (TIF) [file pone.0058157.s001.tif]

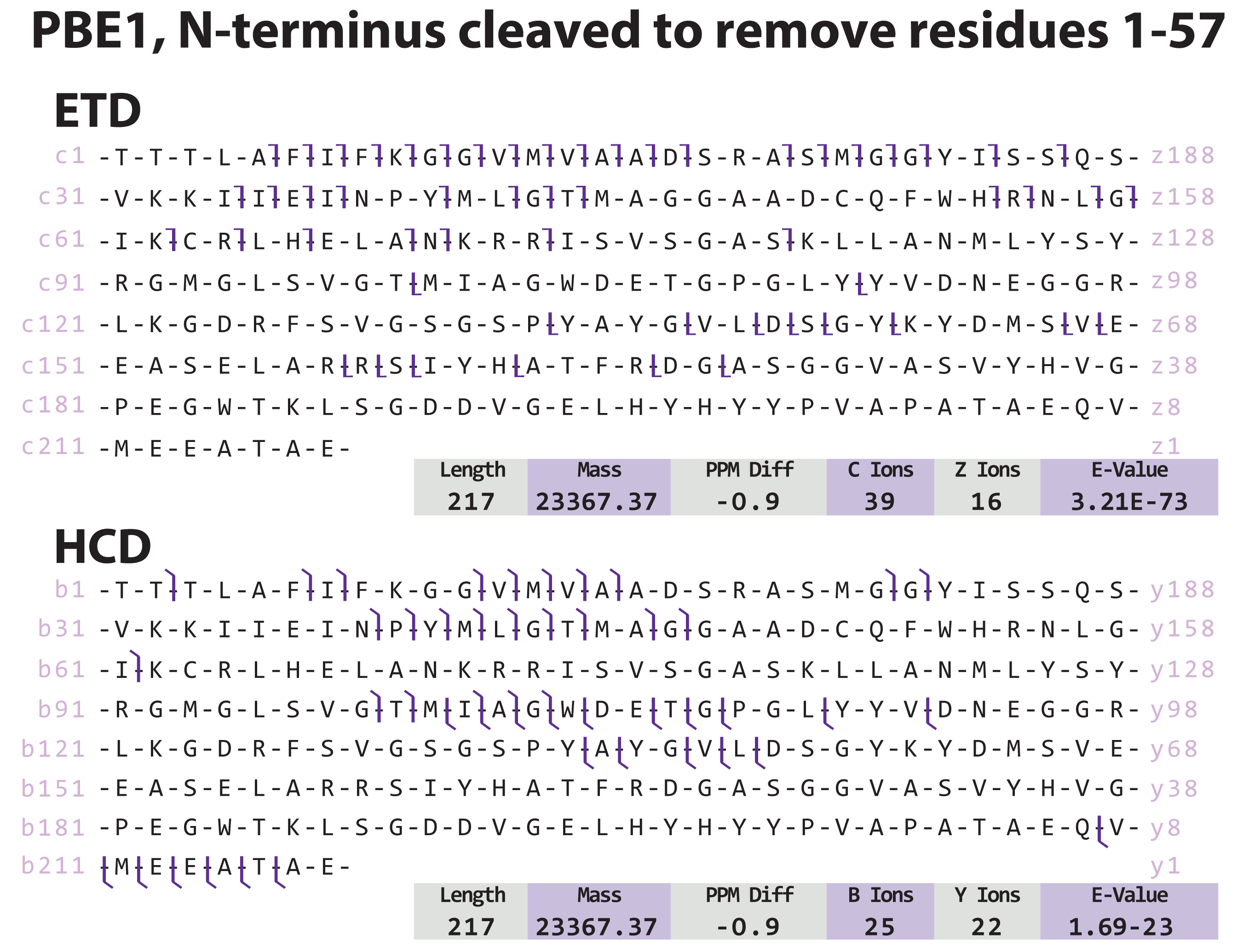

Supplement: Figure S2 — Top-down MS/MS analysis of PBE1. Fragment ion maps for ETD and HCD fragmentation of the N-terminally processed subunit PBE1 (PSB5A, O23717). This modified form was identified with -0.9 ppm mass error and 38% sequence coverage. (TIF) [file pone.0058157.s002.tif]

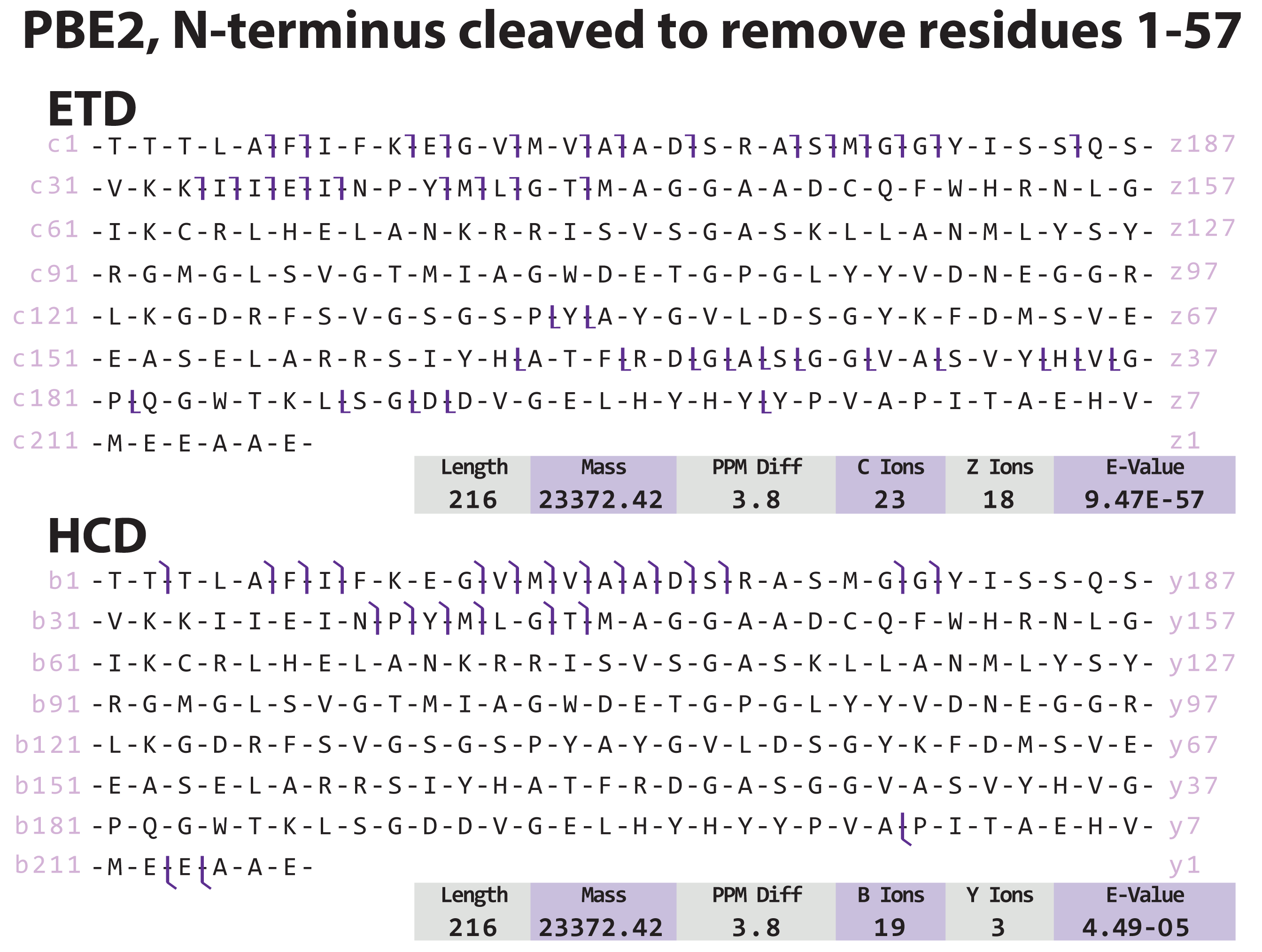

Supplement: Figure S3 — Top-down MS/MS analysis of PBE2. Fragment ion maps for ETD and HCD fragmentation of the N-terminally processed subunit PBE2 (PSB5B, Q9LIP2). This modified form was identified with 3.8 ppm mass error and 25% sequence coverage. (TIF) [file pone.0058157.s003.tif]

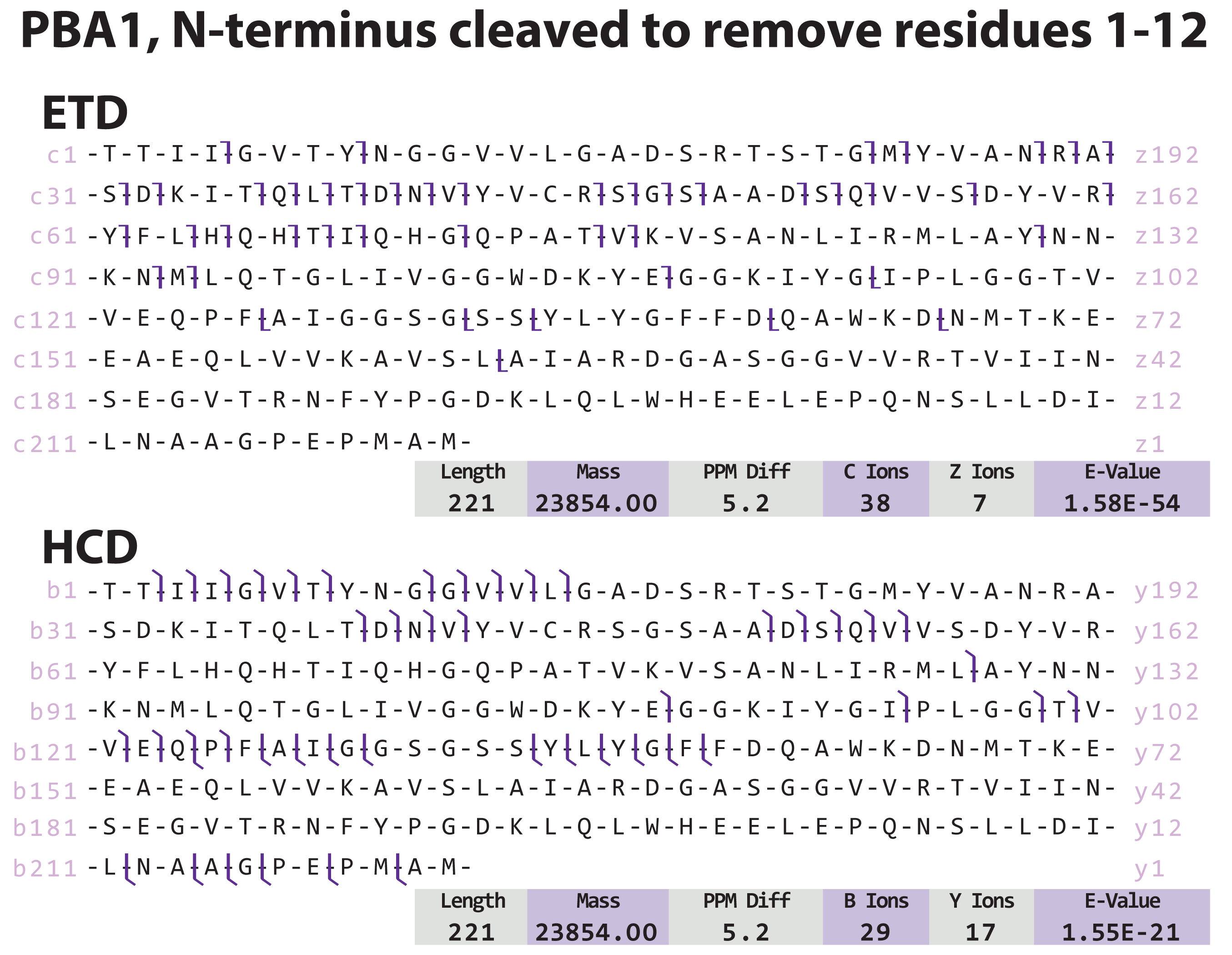

Supplement: Figure S4 — Top-down MS/MS analysis of PBA1. Fragment ion maps for ETD and HCD fragmentation of PBA1 (PSB6, Q8LD27) suggesting N-terminal processing of the first 12 residues. This modified form was identified with 5.2 ppm mass error and 36% sequence coverage. (TIF) [file pone.0058157.s004.tif]

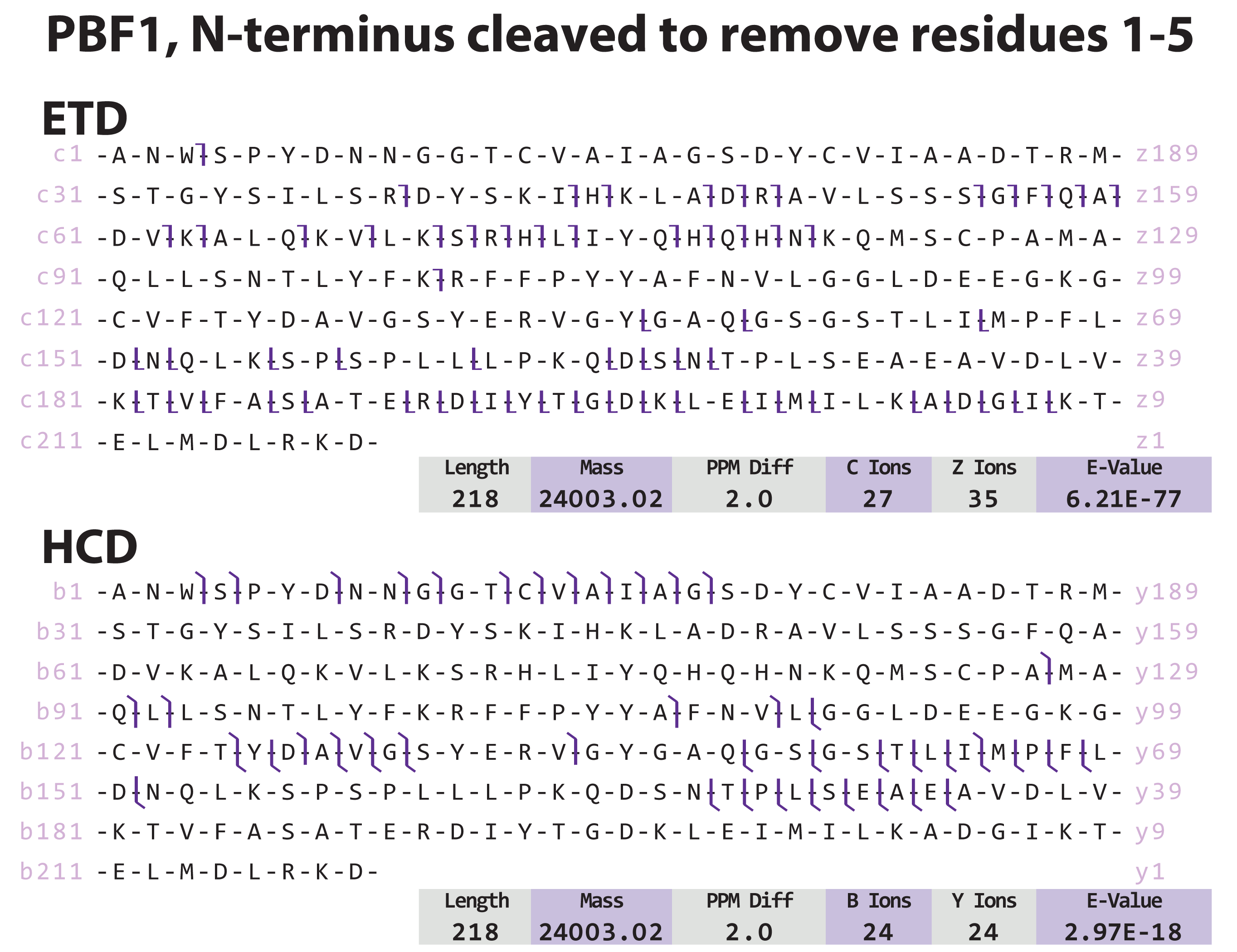

Supplement: Figure S5 — Top-down MS/MS analysis of PBF1. Fragment ion maps for ETD and HCD fragmentation of PBF1 (PSB1, P42742) suggesting N-terminal processing of the first 5 residues. This modified form was identified with 2.0 ppm mass error and 46% sequence coverage. (TIF) [file pone.0058157.s005.tif]

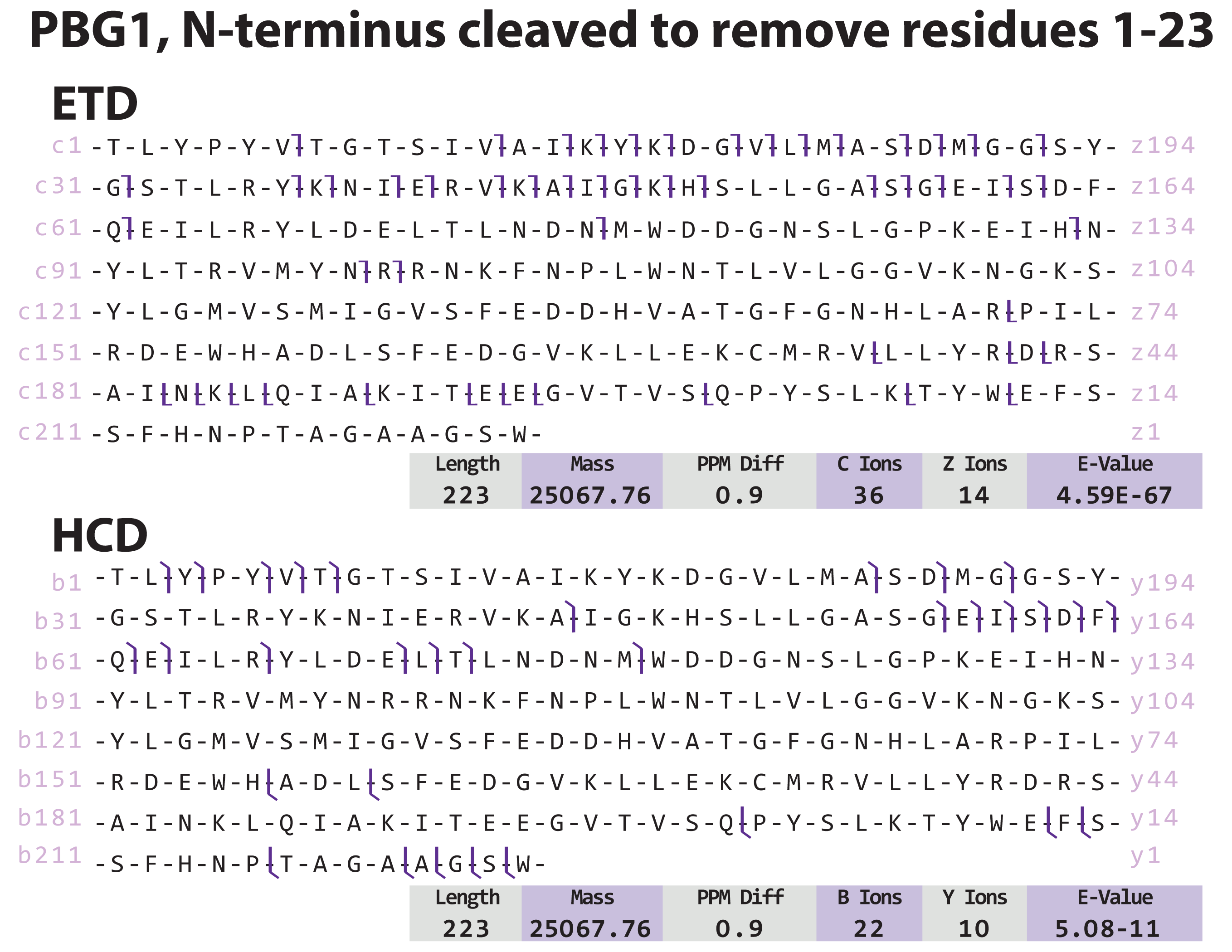

Supplement: Figure S6 — Top-down MS/MS analysis of PBG1. Fragment ion maps for ETD and HCD fragmentation of PBG1 (PSB4, Q7DLR9) suggesting N-terminal processing of the first 23 residues. This modified form was identified with 0.9 ppm mass error and 34% sequence coverage. (TIF) [file pone.0058157.s006.tif]

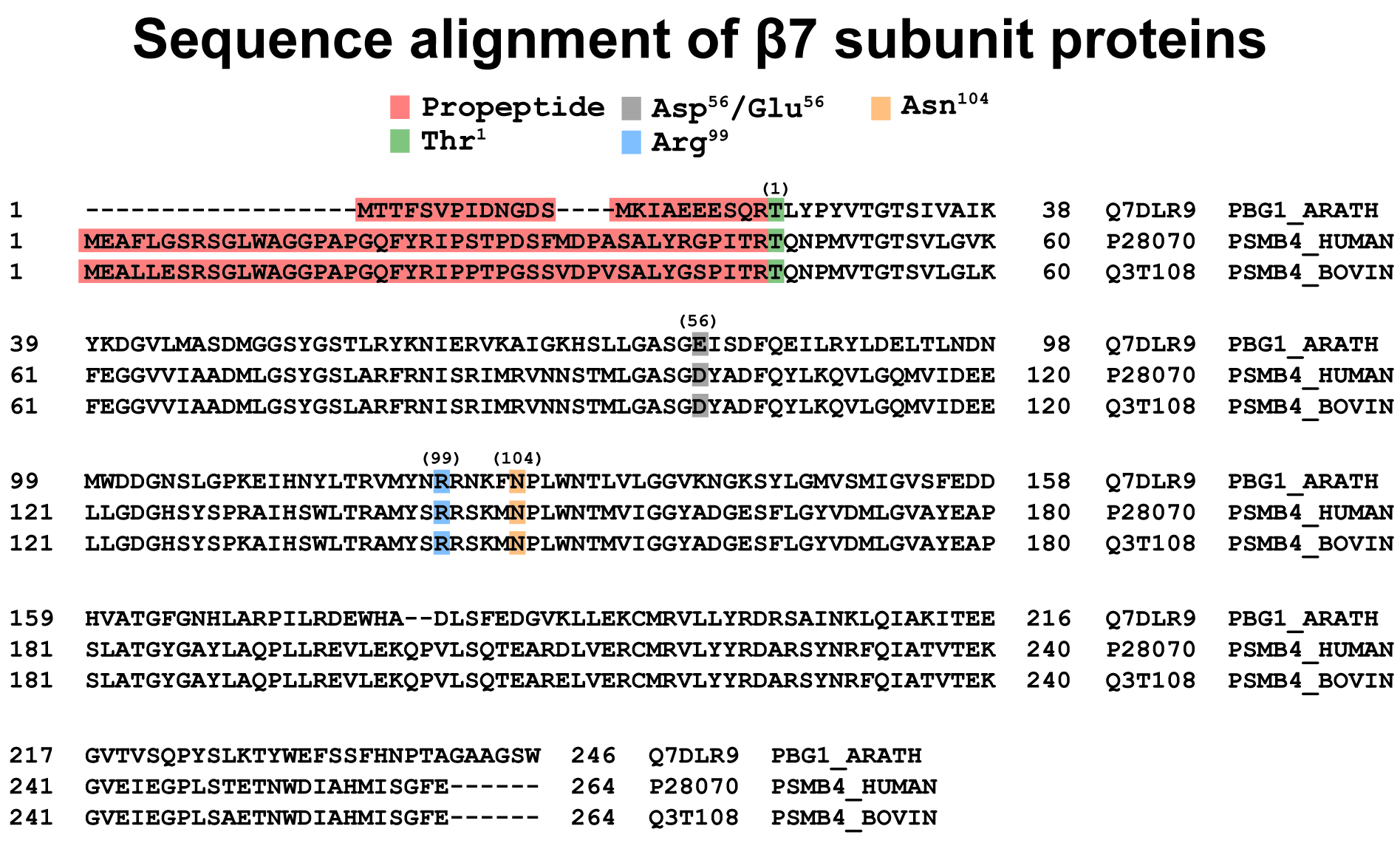

Supplement: Figure S7 — Sequence alignment of Arabidopsis, human, and bovine β7 subunits of the 20 S CP. The sequence alignment was performed using the Align program from UniProt on the 20S CP β7 subunits of Arabidopsis (PBG1, Q7DLR9), human (PSMB4, P28070), and bovine (PSMB4, QT3108) species. All three species release propeptides exposing an N-terminal threonine residue and alignment reveals conserved residues at the 56th, 99th, and 101st positions (relative to Thr1) except for the substitution of Glu for Asp at position 56 for Arabidopsis. These 4 positions are believed to be important for potential proteolytic activity of the β7 subunit [61]. (TIF) [file pone.0058157.s007.tif]

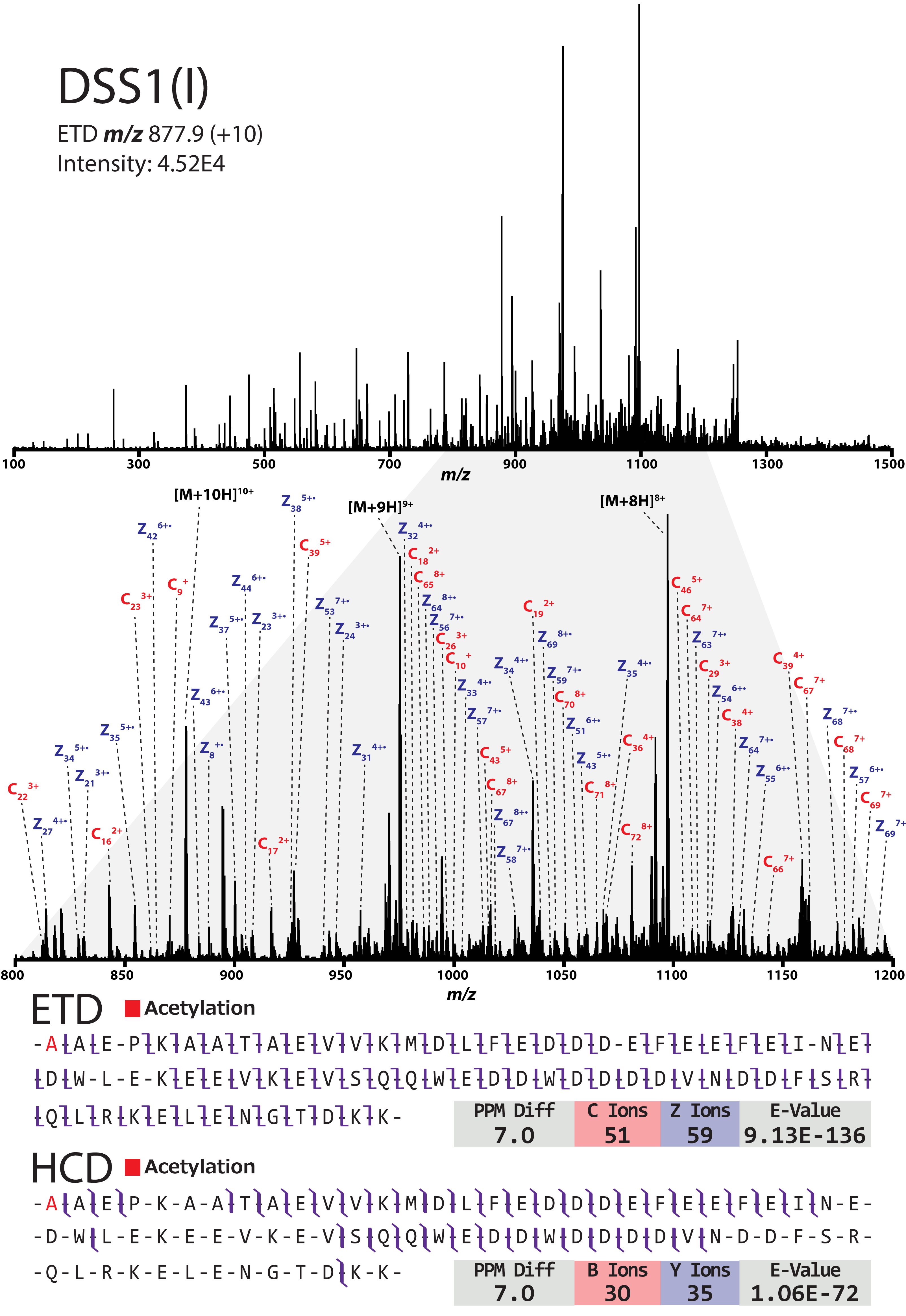

Supplement: Figure S8 — Top-down MS/MS analysis of DSS1(V). ETD spectrum of the proteasome-associated protein DSS1(I) (DSS1-1) and sequence coverage for back-to-back ETD/HCD MS/MS scans. The protein was identified as acetylated on the N-terminal alanine residue. Sequence coverage and mass accuracy unambiguously identify DSS1(I) from the closely related DSS1(V) protein. (TIF) [file pone.0058157.s008.tif]
